# Exotic Rickettsiae *in Ixodes ricinus*: fact or artifact?

**DOI:** 10.1186/1756-3305-3-54

**Published:** 2010-06-22

**Authors:** Ellen Tijsse-Klasen, Manoj Fonville, Leo van Overbeek, Johan HJ Reimerink, Hein Sprong

**Affiliations:** 1 Laboratory for Zoonoses and Environmental Microbiology, National Institute for Public Health and Environment (RIVM), Antonie van Leeuwenhoeklaan 9, P.O. Box 1, Bilthoven, the Netherlands; 2 Wageningen University and Research Centre, Plant Sciences Group, PB, Wageningen, The Netherlands; 3 Laboratory for Infectious Diseases and Screening, National Institute for Public Health and Environment (RIVM), Antonie van Leeuwenhoeklaan 9, P.O. Box 1, Bilthoven, The Netherlands

## Abstract

Several pathogenic *Rickettsia *species can be transmitted via *Ixodes ricinus *ticks to humans and animals. Surveys of *I. ricinus *for the presence of Rickettsiae using part of its 16S rRNA gene yield a plethora of new and different *Rickettsia *sequences. Interpreting these data is sometimes difficult and presenting these findings as new or potentially pathogenic Rickettsiae should be done with caution: a recent report suggested presence of a known human pathogen, *R. australis*, in questing *I. ricinus *ticks in Europe. A refined analysis of these results revealed that *R. helvetica *was most likely to be misinterpreted as *R. australis*. Evidence in the literature is accumulating that rickettsial DNA sequences found in tick lysates can also be derived from other sources than viable, pathogenic Rickettsiae. For example, from endosymbionts, environmental contamination or even horizontal gene transfer.

## Findings

Hard ticks (*Ixodidae*) are the main vectors of the spotted fever Rickettsiae in humans, a group of diseases that is be caused by approximately 20 different *Rickettsia *species of which one-third circulate in Europe [1]. Recently, van Overbeek and colleagues reported the presence of *Rickettsia australis *in *I. ricinus *ticks [2]. They successfully amplified the 16S rRNA gene from DNA extracts from ticks derived from three natural areas in the Netherlands. Sequences of some fragments matched best with *R. australis *(99% similarity), and therefore the authors concluded that they identified *R. australis*. They found 28 ticks positive for the sequence, and reported that the prevalence of *R. australis *varied between 6.7 and 33% in the investigated areas. This finding struck us as remarkable as it would be the first description of this pathogen in Europe. *R. australis *is the etiologic agent of Queensland tick typhus, a disease that normally occurs along the eastern coast of Australia, including Queensland. Clinical features that may be present include fever, headache, myalgia and an eschar at the site of the tick bite. A maculopapular or vesicular skin rash is frequently noted. Patients usually make an uncomplicated recovery, but severe to fatal outcomes have been attributed to *R. australis *infection as well [3]. Thus, the presence of *R. australis *in Europe could have significant public health consequences.

This publication was noted by Lyme patients' associations in The Netherlands and they promptly discussed these findings on their websites (http://www.borreliose.nl/; http://www.lymevereniging.nl/). At our institute, ticks from various sources are routinely tested for potential pathogens [4-7], but during the last decade, we never detected *R. australis-*positive ticks. *R. helvetica*, on the other hand, was found in ticks from every studied location with a prevalence varying from 6 to 67% (~ 6000 ticks). This raised the question how this obvious discrepancy between our results could be explained.

**Figure 1:**
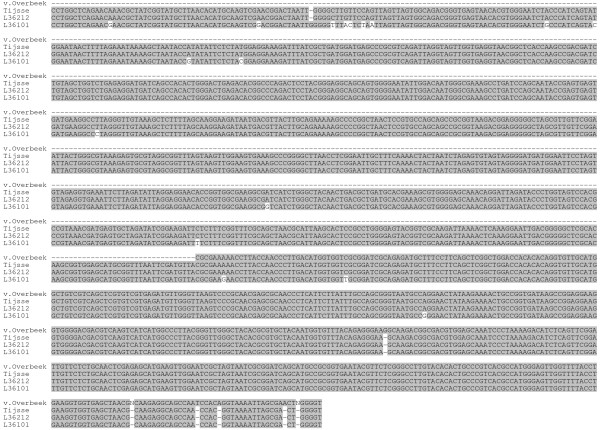
**Comparison of sequences from tick lysates with reference sequence**. 16S rRNA gene sequences from Genbank (*R. australis*: L36101, *R. helvetica*: L36212) were aligned with sequences from the Van Overbeek study (v.Overbeek) and with the sequence we obtained from their samples (Tijsse).

We therefore re-examined the original sequence data of the van Overbeek study, which covered the last 450 bp of the 16S rRNA gene by comparison to sequences of *R. helvetica *[GenBank:L36212] and *R. australis *[GenBank:L36101] published in GenBank (Figure 1). These sequences contained 4 and 7 nucleotide changes compared to the matching target sequences of *R. helvetica *and *R. australis*, respectively. Next, a fraction of the DNA samples of ticks from their study were re-examined with our PCR-amplification protocol followed by Reverse Line Blot (RLB) as described earlier [7]. In theory, our PCR protocol using generic 16S rRNA primers for Rickettsiae would be able to amplify *R. australis *DNA (not shown). The PCR products of these samples hybridized with the *R. helvetica *RLB probe. The PCR-products were sequenced on both strands, and turned out to be 100% identical to the published *R. helvetica *([GenBank:L36212]; Figure 1). For confirmation, part of the *ompB *gene was amplified (729 bp) as an independent marker and sequenced. The sequence was 100% identical to the corresponding *ompB *gene sequence of *R. helvetica *[GenBank:AF123725] and was only 38.9% similar to *R. australis *[GenBank:AF123709]. Thus, we could not detect *R. australis *in the DNA samples of the previous study. Instead, we have strong indications that *R. helvetica *was misidentified as *R. australis *because of a combination of sequencing errors and usage of a limited set of reference sequences.

## Competing interests

The authors declare that they have no competing interests.

## Authors' contributions

ETK and HS performed (bioinformatics) analyses. MF and ETK collected new data. ETK and JHJR wrote the initial draft. HS wrote the final manuscript. LO provided tick lysates and sequence data. JHJR and HS acquired funding. All authors read and approved the final manuscript.
